# Antiseizure Medications: Advancements, Challenges, and Prospects in Drug Development

**DOI:** 10.2174/011570159X323666241029171256

**Published:** 2025-01-24

**Authors:** Yan Hong Ng, Siti Nur Hidayah Jamil, Murni Nazira Sarian, Qamar Uddin Ahmed, Jalifah Latip, Su Datt Lam, Shevin Rizal Feroz

**Affiliations:** 1 Department of Biological Sciences and Biotechnology, Faculty of Science and Technology, Universiti Kebangsaan Malaysia, 43600 Bangi, Selangor, Malaysia;; 2 Department of Chemical Sciences, Faculty of Science and Technology, Universiti Kebangsaan Malaysia, 43600 Bangi, Selangor, Malaysia;; 3 International Joint Department of Materials Science and Engineering Between National University of Malaysia and Gifu University, Graduate School of Engineering, Gifu University, 1-1 Yanagido, 501-1193 Gifu, Japan;; 4 Institute of Systems Biology (INBIOSIS), Universiti Kebangsaan Malaysia, 43600 Bangi, Selangor, Malaysia;; 5 Department of Pharmaceutical Chemistry, Kulliyyah of Pharmacy, International Islamic University Malaysia, 25200 Kuantan, Pahang, Malaysia;; 6 Smart Material and Sustainable Product Innovation (SMatSPIn) Research, Universiti Kebangsaan Malaysia, 43600 Bangi, Selangor, Malaysia;; 7 Department of Applied Physics, Faculty of Science and Technology, Universiti Kebangsaan Malaysia, 43600 Bangi, Selangor, Malaysia;; 8 Structural Biology and Protein Engineering Research Group, Universiti Kebangsaan Malaysia, 43600 Bangi, Selangor, Malaysia;; 9 Center for Global Health Research (CGHR), Saveetha Medical College, Saveetha Institute of Medical and Technical Sciences (SIMATS), Saveetha University, 602105 Chennai, India

**Keywords:** Epilepsy, antiseizure medications, γ-aminobutyric acid type A receptor, isoguvacine, neurotransmission, neurological disorder

## Abstract

Epilepsy is a neurological disorder affecting millions of people worldwide. Antiseizure medications (ASM) remain a critical therapeutic intervention for treating epilepsy, notwithstanding the rapid development of other therapies. There have been substantial advances in epilepsy medications over the past three decades, with over 20 ASMs now available commercially. Here we describe the conventional and unique mechanisms of action of ASMs, focusing on everolimus, cannabidiol, cenobamate, fenfluramine, and ganaxolone, the five most recently marketed ASMs. Major obstacles in the development of ASMs are also addressed, particularly drug-resistant epilepsy as well as psychiatric and behavioral adverse effects of ASMs. Moreover, we delve into the mechanisms and comparative efficacy of ASM polytherapy, with remarks on the benefits and challenges in their application in clinical practice. In addition, the characteristics of the ideal ASM are outlined in this review. The review also discusses the development of new potential ASMs, including modifying existing ASMs to improve efficacy and tolerability. Furthermore, we expound on the modulation of γ-aminobutyric acid type A receptor (GABA_A_R) as a strategy for the treatment of epilepsy and the identification of a GABA_A_R agonist, isoguvacine, as a potential ASM.

## 1 INTRODUCTION

Epilepsy has been described in medical records since as early as 4000 BCE and is one of the most common neurological disorders characterized by recurrent seizures resulting from abnormal electrical activity in the brain [1]. These seizures can vary in intensity, duration, and manifestation, leading to significant disruptions in the daily lives of those affected [2]. The estimated worldwide prevalence of epilepsy is approximately 7 cases per 1,000 individuals, totaling 5 million new cases annually, with an overall estimate of 50 million people living with epilepsy. Although epilepsy is a global health concern, around 80% of all patients are concentrated in rural areas of low- and middle-income countries [3].

In 2022, the US National Institute of Neurological Disorders and Stroke (NINDS) and the Interagency Collaborative to Advance Research in Epilepsy (ICARE) allocated USD 212 million for epilepsy research, focusing on antiepileptic treatments. Additionally, the World Health Organization (WHO) launched the “Intersectoral Global Action Plan on epilepsy and Other Neurological Disorders 2022-2031” during the 75^th^ World Health Assembly, addressing the epilepsy burden through pharmacological and psychosocial strategies as outlined in the epilepsy technical brief published in 2022 [4].

Antiseizure medications (ASMs) are crucial in managing epilepsy by regulating the balance between excitation and inhibition in neurotransmission. They control the occurrence and propagation of seizures by effectively regulating electrical activity within the brain [5]. Continued advancements in epilepsy treatment have necessitated a change in terminology to accurately describe the effects of epilepsy therapies. It is worth acknowledging here the evolution of ASMs, formerly referred to as anticonvulsants or antiepileptic drugs. The substitution of the term “anticonvulsant drug” with “antiseizure medication” stems from its ability to manage both convulsive and nonconvulsive seizures. This shift is preferred over “antiepileptic drug” due to ASMs' unique characteristic of providing relief without altering the overall trajectory of epilepsy [6]. Using the term “antiseizure medication” accurately describes the primary effect of these drugs, preventing misunderstandings and unrealistic expectations. Consequently, it is crucial to recognize that their application in treating epilepsy and related conditions relies solely on the suppression of seizures [7].

While ASMs are effective in controlling seizures for many individuals, they are however not a cure for epilepsy. In general, ASMs have been proven to effectively control seizures in 60 to 70% of treated epilepsy cases [8]. Despite the development of new ASMs, a third of patients are said to have drug-resistant epilepsy, where they remained refractory to treatment even when adequate treatment was provided and the patients adhered to medication [9]. Additionally, like any other medications, ASMs can have side effects, which can range from mild to severe depending on the drug and the individual's response. It was reported that the administration of existing ASMs has led to psychiatric and behavioral side effects in about 20% of adult patients [10]. Thus, regular monitoring and communication with healthcare professionals are crucial to ensure optimal seizure control and minimize adverse effects [11].

Access to appropriate epilepsy treatment remains a challenge despite the increasing availability of ASMs. While ASMs have improved seizure management, not all patients respond well, and adherence can be challenging due to factors such as side effects and cost. In this review, we describe the various mechanisms of action underlying ASMs and highlight the prevailing challenges in epilepsy treatment. We also discuss the pressing need for the development of novel ASMs, while exploring the characteristics of an ideal ASM and several promising drug candidates for epilepsy. Thus, this work is expected to provide valuable insights into the future landscape of epilepsy therapy while carefully considering the potential impacts of novel antiepileptic medications on seizure threshold and their side effects.

## 2 MECHANISMS OF ACTION OF ANTISEIZURE MEDICATIONS

The various excitatory and inhibitory neurotransmitters are crucial for communication between neurons and the brain's normal functioning. An imbalance in the levels of these neurotransmitters has been identified as one of the main causes of the occurrence of seizures. ASMs are important in the treatment of epilepsy by modulating neurotransmission through the targeting of ion channels, receptors, and synaptic activity involving neurons and glial cells. A comprehensive understanding of the diverse mechanisms of action of ASMs is crucial for their effective use, as summarized in Fig. (**1**).

### 2.1 Modulation of Voltage-Gated Ion Channels

Voltage-gated ion channels are a class of transmembrane proteins involved in the generation and propagation of action potentials by controlling the flow of ions in response to changes in membrane potential. These channels are ion-specific, such as sodium, calcium, potassium, and chloride, and they have a similar mechanism of activation. Due to their roles in the generation and propagation of action potentials in neurons and other cells, they are common therapeutic targets for many ASMs. Ion channels that belong to the voltage-gated ion channel superfamily share several common structural features as illustrated in Fig. (**2**). The α-subunit forms the core of the channel, composed of four homologous domains (D1-D4), each containing six transmembrane segments (S1-S6). The S1-S4 segments constitute the voltage-sensing module, while the pore loops between the S5 and S6 segments act as the selectivity filter [12]. The activation gate for these channels is located at the intracellular end of the S6 segments within the pore-forming α subunit [13, 14]. Voltage-gated sodium and calcium channels have an additional inactivation gate positioned within the intracellular loop between D3 and D4 [15]. These gating mechanisms are crucial for regulating ion flux and cellular excitability in response to changes in membrane potential. Despite these shared features, voltage-gated ion channels vary significantly in ion conductance, activation and inactivation mechanisms, ion selectivity, and modulation by auxiliary subunits, reflecting their distinct cellular functions and physiological roles.

#### 2.1.1 Modulation of Voltage-gated Sodium Channels

Voltage-gated sodium channels (VGSCs) are integral membrane proteins that are responsible for regulating the electrical signaling of cells. They are mostly found in neuronal, neuroendocrine, skeletal muscle, and cardiac cells [16]. VGSCs are a highly glycosylated protein complex that consists of a large α subunit (260 kDa) and two auxiliary β-subunits (about 35 kDa). The activation gate of the channel, found at the intracellular end of the S6 segments [17, 18], allows for the binding of pore-blocking molecules such as local anesthetics, antiarrhythmic drugs, and ASMs that block the channel in a voltage-dependent manner [19].

Theoretically, VGSCs may exist in three states, which are open, closed, and inactive. At normal membrane potentials, the sodium channels are in a closed and resting state. However, upon depolarization, the channels are activated and undergo a physical conformational change that allows a transient influx of sodium ions into the cell to initiate the rising phase of the action potential. As the membrane potential becomes positive, the sodium influx is reduced, and the channels begin to close. The channels remain inactive in the refractory period and revert to the resting state when the membrane is repolarized. Hence, VGSCs undergo rapid activation, followed by fast and slow inactivation processes [20].

ASMs that target VGSCs, such as carbamazepine, lacosamide, and phenytoin, are frequently used to treat partial and generalized tonic-clonic seizures because they stabilize channel inactivation and prevent recurrent neuronal firing [21-23]. Similar in its mode of action, valproate is a more potent anticonvulsant [24]. More recently developed ASMs that block sodium channels such as topiramate, zonisamide, oxcarbazepine, lamotrigine, felbamate, and rufinamide, work by targeting the fast inactivation mechanism [16, 25].

The action potential upstroke is mediated by the transient voltage-gated sodium current. However, a small fraction of sodium current, known as the persistent sodium current (INaP), remains even after extended depolarization. This current is active in the subthreshold voltage range and can modify cell firing and promote hyperexcitability, such as in a seizure event [26]. On the other hand, a growing body of research attributes the occurrence of epilepsy to mutations in sodium channels, some of which increase INaP [27]. Cenobamate, a new ASM, approved for clinical use by the US Food and Drug Administration (FDA) and the European Medicines Agency (EMA) in 2021 and 2019, respectively [28], has been shown to reduce INaP, promotes a more potentiated inactivation state of the sodium channel, and act as a positive allosteric GABA_A_R modulator [6].

#### 2.1.2 Modulation of Voltage-gated Calcium Channels

Voltage-gated calcium channels (VGCCs) are transmembrane proteins that allow the entry of calcium ions during membrane depolarization, which in turn, further depolarizes the plasma membrane [29]. The calcium ions that enter cells through VGCCs also act as second messengers of cellular signaling, leading to the initiation of various downstream responses. These calcium channels play critical roles in cellular physiology, serving as regulators of muscle contraction, neurotransmitter release, gene expression, and various other physiological processes [30]. VGCCs consist of several subunits forming a complex structure. The general structure of the α1 subunit, the main ion-conducting pore, is similar to that of VGSCs, as shown in Fig. (**2**). The presence of auxiliary β subunits (β1, β2, β3, or β4) modulates channel function and membrane trafficking, while the α2δ complex (comprising individual α2 and δ subunits) enhances channel activity and facilitates interactions with other proteins. Some VGCCs may also have γ subunits (stargazin), involved in channel trafficking, synaptic targeting, and function modulation [31].

VGCCs are broadly classified into high voltage-activated (HVA) and low voltage-activated (LVA) channels [32]. The LVA T-type calcium channels, in particular, are recognized for their ability to regulate neuronal firing by participating in bursting and intrinsic oscillations [33]. These calcium channels are highly significant in the pathophysiology of absence epilepsy, characterized by spike-and-wave discharges. Specifically, these channels are involved in generating low threshold spikes, which contribute to the burst firing of thalamic relay neurons [34, 35].

**Fig. (3):**
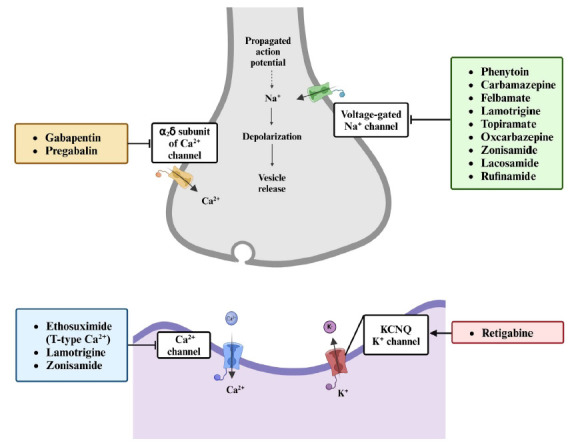
Mechanism of action of commonly prescribed ASMs that modulate the activity of voltage-gated ion channels, such as the sodium (Na^+^), calcium (Ca^2+^), and potassium (K^+^) channels, to control neuronal excitability in the central nervous system. These ASMs affect neurotransmission by targeting the flow of ions through these channels, which is crucial for the generation and propagation of action potentials. (KCNQ: K_v_7 potassium channel family).

To control the spread of absence seizures, several ASMs have been developed to specifically target and block the T-type calcium channels (Fig. **3**). By interfering with channel activity, these medications can help regulate the abnormal electrical discharges associated with absence epilepsy. Examples of ASMs that have been proven to effectively manage absence seizures are ethosuximide and zonisamide, which act by reducing the activity of low-threshold T-type calcium currents in thalamocortical neurons [36]. Due to its multiple modes of action, zonisamide is widely used for treating a wide range of epilepsies in adults and children, both as monotherapy and as an adjunctive agent [37, 38].

#### 2.1.3 Modulation of Voltage-gated Potassium Channels

Voltage-gated potassium (K_v_) channels are transmembrane proteins that facilitate neuronal excitability in the central and peripheral nervous system. The K_v_ channels in humans are encoded by 40 genes and can be classified into 12 subfamilies (K_v_1 to K_v_12) [39]. Activation of K_v_ by membrane depolarization triggers an outward movement of potassium ions and this leads to repolarization of action potential. Efflux of potassium ions results in hyperpolarization of membrane potential and plays a critical role in establishing resting membrane potential, in addition to regulating neurotransmitter release [40, 41].

Dysfunctions of K_v_, especially K_v_1 and K_v_7, have been associated with hyperexcitability phenotypes such as epilepsy [42, 43]. Consequently, K_v_ channels have become targets in developing ASMs. One such example is retigabine which modulates the K_v_7 channels, specifically the K_v_7.2 to K_v_7.5 subunits (also known as KCNQ2-5) (Fig. **3**). Notably, the KCNQ2/Q3 channels underlie the M current, a non-inactivating potassium current [44]. This current is activated at subthreshold potentials, hyperpolarizes the cell membrane, and reduces the firing of the action potential. Thus, it is suggested that the M current is involved in the control of neuronal excitability [45]. Retigabine acts as a KCNQ potassium channel opener by triggering a shift in voltage-dependent activation of the channel that favors more negative voltages, thus enhancing the activation of KCNQ2/Q3 channels [46].

### 2.2 Enhancement of Inhibitory Neurotransmission

Gamma-aminobutyric acid (GABA) is the most abundant inhibitory neurotransmitter in the vertebrate central nervous system (CNS) that plays a pivotal role in reducing neuronal excitability. GABA is synthesized from glutamate and is metabolized by GABA transaminase (GABA-T) in the GABA shunt. The GABA shunt is an alternative metabolic pathway that bypasses two enzymatic steps of the tricarboxylic acid (TCA) cycle. This pathway initiates with the transamination of α-ketoglutarate to yield glutamate, followed by the decarboxylation of glutamate to γ-aminobutyric acid (GABA) *via* glutamate decarboxylase (GAD). Subsequently, GABA undergoes transamination catalyzed by GABA transaminase (GABA-T), producing succinic semialdehyde. The final step involves the oxidation of succinic semialdehyde to succinate, which is then reintegrated into the TCA cycle, thus completing the shunt [47]. Disruptions in this pathway can reduce inhibitory neurotransmission and contribute to neuronal hyperexcitability, a characteristic of epilepsy. GABA primarily interacts with two classes of receptors in the CNS, namely GABA_A_ and GABA_B_ receptors. Ionotropic GABA_A_ receptors are ligand-gated chloride channels located in the postsynaptic membrane [48]. Activation of GABA_A_ receptors (GABA_A_R) leads to hyperpolarization of postsynaptic membranes which inhibits signal transmission as the postsynaptic inhibitory potential is induced [49]. Dysfunction of GABA_A_R has been linked to the development of epilepsy and status epilepticus [50].

**Fig. (4):**
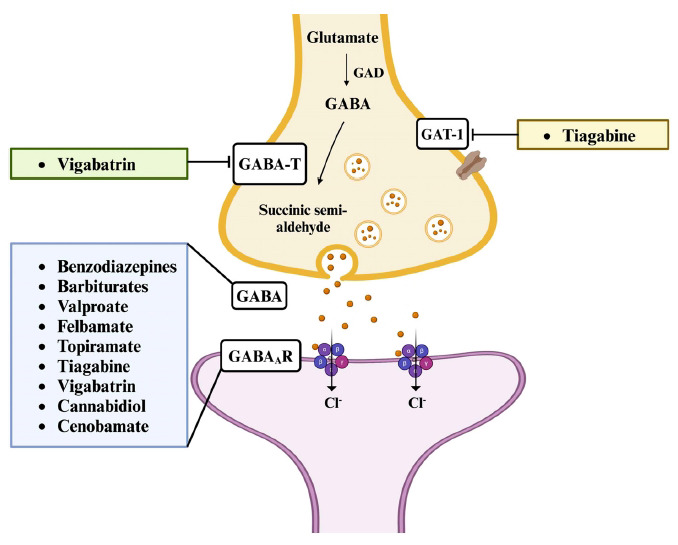
Mechanisms of action of ASMs that promote inhibitory neurotransmission in the CNS. **Abbreviations:** GABA: γ-aminobutyric acid; GABA_A_R: GABA type A receptor; GABA-T: GABA transaminase; GAD: Glutamic acid decarboxylase; GAT-1: GABA transporter 1.

Drugs capable of enhancing GABAergic transmission have been demonstrated to have antiepileptic properties (Fig. **4**). This is primarily achieved by stimulating the inhibitory effect of GABA_A_Rs, making them targets of agonists such as benzodiazepines (lorazepam, diazepam, clonazepam, and clobazam), barbiturates (phenobarbital and primidone), cannabidiol, and loreclezole. Notably, barbiturates can activate these receptors directly even in the absence of GABA, particularly at higher concentrations [51, 52]. ASMs such as felbamate, topiramate, gabapentin, and valproic acid may also promote the inhibitory actions of GABA by increasing GABAergic neurotransmission or by modulating the activity of GABA_A_R [53]. Since the metabolism of GABA by GABA-T would decrease its extracellular concentration, it is thought that the inhibition of this enzyme will prevent the degradation of GABA and thus maintain its inhibitory effects. For example, vigabatrin is a widely used ASM that exerts its therapeutic effects through the inhibition of GABA-T. By binding irreversibly to the active site of the enzyme, vigabatrin enhances GABAergic neurotransmission, resulting in its antiseizure effects [54]. Moreover, inhibition of the reuptake of GABA *via* GABA transporter 1 (GAT-1) by tiagabine, a GABA transport inhibitor, also increases the concentration of GABA in the synaptic cleft [55].

### 2.3 Inhibition of Excitatory Neurotransmission

**Fig. (5):**
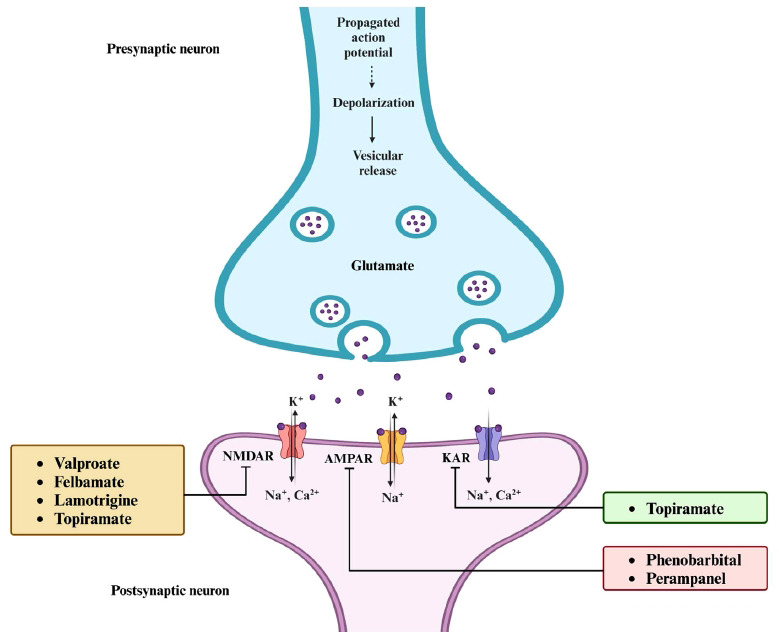
Mechanisms of action of ASMs that act on different receptors to block excitatory neurotransmission in the CNS. **Abbreviations:** AMPAR: α-amino-3-hydroxy-5-methyl-4-isoxazole propionic acid receptor; KAR: kainate receptor; NMDAR: N-methyl-D-aspartate receptor.

Glutamate, the major excitatory neurotransmitter in the CNS, interacts with ionotropic and metabotropic glutamate receptors to induce excitatory neurotransmission [56, 57]. Ionotropic glutamate receptors (iGluRs), which include the N-methyl-D-aspartate (NMDA), α-amino-3-hydroxy-5-methyl-4-isoxazolepropionic acid (AMPA), and kainate receptors, mediate fast excitatory neurotransmission and are pivotal in synaptic plasticity [58]. Metabotropic glutamate receptors are G-protein coupled receptors that engage in the modulation of excitatory neurotransmission *via* intracellular second messengers [59]. These receptors, widely expressed in postsynaptic cell dendrites and glial cells, are implicated in the generation and spread of epileptic seizures [60]. Therefore, the blockade of excitatory neurotransmission *via* these receptors features as another mechanism of action of ASMs (Fig. **5**).

The AMPA receptor, a key player in excitatory postsynaptic signaling, is widely distributed throughout the CNS. Activation of AMPA receptors in the postsynaptic membrane triggers action potentials, facilitating fast and efficient synaptic transmission in the brain [61, 62]. This vital role of AMPA receptors in neural communication has spurred intense research into their involvement in epilepsy and epileptogenesis, with a particular focus on developing novel ASMs targeting these receptors [63].

NMDA receptors are abundant in the cerebral cortex and hippocampus and have important physiological and pathological functions in the CNS. They are mandatory heterotetrameric assemblies that typically consist of two glycine-binding GluN1 subunits and two glutamate-binding GluN2A-D subunits [64, 65]. When glutamate binds to the GluN2 subunits, NMDA receptors are activated. This is followed by membrane depolarization, which releases the Mg^2+^ block on the channel. The activation and opening of this receptor results in the simultaneous inflow of Na^+^ and Ca^2+^ ions and the outflow of K^+^ ions [66, 67].

Kainate receptors are heterotetramers of subtypes GluR1-5, which are found in many regions of the nervous system. Despite having several molecular and physiological traits in common with the AMPA receptors, they possess distinct receptor assemblies [68, 69]. Stimulation of kainate receptors induces a rapid influx of Na^+^ and Ca^2+^ ions, mediating excitatory neurotransmission [70]. Its agonist, kainate, is frequently been used to induce acute brain seizures [71]. These receptors are associated with seizures and epilepsy as the excitatory and inhibitory neurotransmissions in the hippocampus and amygdala are modulated by kainate receptors that comprise the GluK1 subunit [72].

### 2.4 Modulation of SV2A Vesicle Protein

Synaptic vesicle glycoprotein 2A (SV2A), one of the members of the synaptic vesicle proteins 2 family, has emerged as a new molecular target for ASMs. This protein is ubiquitously expressed in the synaptic neurons and endocrine cells [73] and has been proposed to be involved in synaptic vesicle exocytosis and modulation of calcium-dependent neurotransmitter release [74]. Recently, the role of SV2A in the pathogenesis and treatment of epilepsy has gained significant attention due to compelling evidence. Studies using SV2A-knockout mice have revealed that the absence of SV2A leads to severe convulsive seizures, highlighting its involvement in seizure control [75]. Furthermore, alterations in SV2A expression have been observed in various epileptic disorders, both in animal models (such as kindling and genetic models) and in humans (including intractable temporal lobe epilepsy and focal cortical dysplasia) [76].

**Fig. (6):**
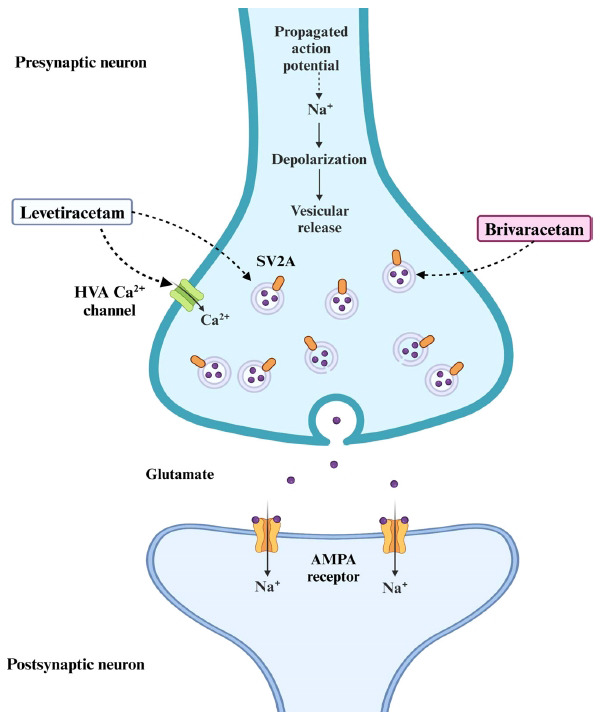
Mechanisms of action of levetiracetam and brivaracetam involving the SV2A vesicle protein leading to reduced neuronal excitability. (AMPA: α-amino-3-hydroxy-5-methyl-4-isoxazole propionic acid; HVA Ca^2+^ channel: high-voltage-activated calcium channel; SV2A: synaptic vesicle protein 2A).

The SV2A vesicle protein serves as a specific binding site for ASMs such as levetiracetam and its analog, brivaracetam, emphasizing its relevance in the treatment of seizures (Fig. **6**). These drugs bind directly to SV2A, resulting in the reduced release of neurotransmitters. While SV2A is involved in the release of both excitatory and inhibitory neurotransmitters, the net effect of SV2A modulation by these drugs appears to be a reduction in neuronal excitability. This reduction may occur through complex mechanisms involving the balance between neurotransmitter release, potential alterations in receptor sensitivity, or interactions with other synaptic proteins [77, 78]. Additionally, the interaction of levetiracetam with SV2A is believed to stabilize synaptic vesicle fusion, preventing excessive glutamate release and thereby maintaining neuronal homeostasis during periods of heightened neuronal activity [78]. Furthermore, levetiracetam also inhibits high-voltage-activated (HVA) calcium channels, reducing calcium influx and further dampening neuronal excitability [79]. This dual mechanism-SV2A binding and HVA calcium channel inhibition-underscores the multifaceted approach of levetiracetam in controlling seizures. Although it is unclear how SV2A modulation decreases neuronal excitability, it is thought to involve the maintenance of synaptic vesicle availability and the regulation of synaptic responsiveness to neuronal activity. Collectively, these insights support the notion that SV2A plays a significant role in both the pathogenesis and treatment of epilepsy, making it a promising target for future therapeutic interventions.

### 2.5 Inhibition of mTOR

The mechanistic target of rapamycin (mTOR) is a serine/threonine kinase in the phosphatidylinositol 3-kinase (PI3K)-related kinases (PIKK) family [80]. As a key player in cell and organismal physiology, the mTOR protein kinase triggers a signaling network that responds to environmental factors such as nutrient availability, growth factors, oxygen levels, energy status, stress, hormones, and environmental toxins, thus regulating the growth and development of cells [81, 82]. Involved in several different signaling pathways throughout the body, mTOR controls transcription, autophagy, protein synthesis, cell motility, growth, and other cellular processes [83]. The mTOR complex 1 (mTORC1) and mTOR complex 2 (mTORC2) are two structurally distinct complexes that incorporate the mTOR protein. These protein complexes are located in different subcellular compartments, possess discrete protein components, and phosphorylate a variety of substrates [84]. While mTORC1 is a rapamycin-sensitive complex that controls cell growth and metabolism, mTORC2 is a rapamycin-insensitive complex that controls cell survival, proliferation, and migration as well as cytoskeletal remodeling [85, 86].

Numerous disorders, including epilepsy, tuberous sclerosis complex (TSC), and cancer, are impacted by mTOR dysregulation [87]. Studies have shown that mTOR signaling is commonly overactivated in epilepsy, indicating that this pathway may be significant in the pathophysiology and therapeutic management of epilepsy [88]. While epilepsy is characterized by abnormal neuronal electrical activity, mTOR overactivation contributes to this through multiple mechanisms. These include altering synaptic plasticity, increasing neuronal excitability, and promoting abnormal neuronal growth and connectivity [89]. This hyperactivation leads to increased protein synthesis of ion channels and neurotransmitter receptors, altering the electrical properties of neurons. Furthermore, mTOR-mediated autophagy failure can cause aberrations in the clustering of GABA_A_Rs at synapses, leading to an imbalance between excitation and inhibition-a key factor in epileptogenesis [90]. mTOR hyperactivation also suppresses autophagy, which normally regulates processes like axon guidance, synapse development, and synaptic plasticity. This suppression can result in the accumulation of NMDA receptor and AMPA glutamate receptors, leading to irregular glutamate signaling and calcium influx [91].

**Fig. (7):**
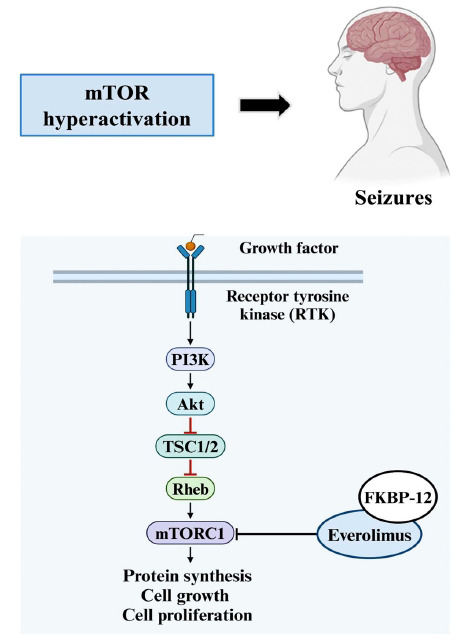
Mechanism of action of everolimus in regulating hyperactivation of the mTOR pathway in TSC that leads to seizures. (Akt: Protein kinase B; FKBP-12: FK506-binding protein 12; mTORC1: Mechanistic target of rapamycin complex 1; PI3K: Phosphoinositide 3-kinase; Rheb: Ras homolog enriched in brain; TSC1/2: Tuberous sclerosis complex 1 and 2).

Additionally, mTOR inhibitors have been demonstrated to be beneficial in treating a range of experimental forms of hereditary and acquired epilepsy when mTOR hyperactivation is present [92]. Thus, pharmaceutical targeting of mTOR inhibitors for epilepsy and epileptogenesis is a topic of growing interest. Everolimus, for example, is a specific inhibitor of the mTOR pathway that was authorized by the FDA in 2018 to treat TSC-induced partial-onset seizures by blocking mTOR activity and immune cell proliferation (Fig. **7**) [93]. The mTOR pathway is initiated by PI3K, which activates PDK1 and subsequently Akt. Akt inhibits the TSC1/2 protein complex by phosphorylation. Usually, TSC1/2 acts as a negative regulator of the mTOR pathway by inhibiting Rheb. However, mutations in TSC1/2 disrupt this inhibition, resulting in mTOR hyperactivation. Everolimus, in complex with FKBP-12, interacts with and inhibits mTOR, thus attenuating mTORC1 activity. Inhibition of mTORC1 by everolimus thus decreases the phosphorylation of 4E-BP1 (eukaryotic translation initiation factor 4E-binding protein 1) and S6K ½ (ribosomal protein S6 kinase 1/2), regulating protein synthesis and cell growth.

## 3 DEVELOPMENT OF ANTISEIZURE MEDICATIONS

### 3.1 From Past to Present

Antiseizure medications are pharmaceuticals employed in managing epilepsy, with a history tracing back to the 19^th^ century. Potassium bromide (KBr), the first anti-seizure medication, was introduced in 1857 by Sir Charles Locock. Initially, KBr was used empirically, as its anticonvulsant mechanisms were not understood at the time. Subsequent research has revealed that its effects are primarily attributed to bromide ions, which cross cell membranes and inhibit neuronal excitability, potentially impacting epileptic activity beyond synaptic modulation [94]. Although the specific molecular targets of KBr remain poorly defined, necessitating further research to elucidate its precise mechanisms of action, its initial effectiveness as an anticonvulsant is well-documented. However, the use of KBr has declined over time due to its narrow therapeutic index, undesirable side effects, and the development of safer and more effective anti-epileptic drugs [95]. Additionally, its long half-life and the potential for accumulation in the body increase the risk of toxicity [96]. In the early 20^th^ century, phenobarbitone and phenytoin emerged as primary ASMs in the treatment of epilepsy. The discovery of more modern ASMs was aided by advancements made since the 1960s, particularly a better understanding of the electrochemical activity of the brain and its neurotransmitters [97].

The development of ASMs has seen significant advancements over the years, resulting in medications with distinct mechanisms of action and varying effectiveness in seizure control. Beyond epilepsy, ASMs may also be prescribed for other neurological conditions, including neuropathic pain, bipolar disorder, and migraine prophylaxis. Some ASMs possess mood-stabilizing properties and can help manage certain types of pain, making them useful in the treatment of these conditions [98]. Selection depends on factors such as type of epilepsy, age of the patient, preexisting conditions, and potential drug interactions [99]. ASMs come in various formulations such as tablets, capsules, oral solutions, extended-release forms, and injectables [100, 101]. The choice depends on patient compliance and dosing frequency, with dosing adjusted based on patient response and side effects [102].

Between 1912 and 2022, a total of 28 ASMs received FDA approval for treating epilepsy as outlined in Table (**1**). The classification of ASMs into three generations is determined by the chronological order of their development, rather than by structural or mechanistic characteristics. These generations reflect the evolution of ASMs over time and encompass medications developed during specific periods. This classification helps to contextualize the historical progression of epilepsy treatment options, allowing for a clearer understanding of the advancements made in the field. The development of ASMs has progressed through three key generations, with each trying to improve upon its predecessor in terms of efficacy, safety, and pharmacokinetics. However, certain limitations and challenges remain, which require a deeper understanding of the pathogenesis of epilepsy and *in vivo* drug behavior to overcome.

#### 3.1.1 First Generation (1912-1981)

First-generation ASMs were developed as early as 1912, encompassing medications like phenytoin, carbamazepine, valproic acid, and phenobarbital. Despite their effectiveness in managing seizures, there are some notable drawbacks associated with first-generation ASMs. For example, phenytoin and valproic acid have high plasma protein binding (≥ 90%), which reduces the concentration of the unbound drug in the bloodstream, thus attenuating their efficacy [103]. Phenytoin also follows zero-order kinetics, meaning that its elimination rate is constant regardless of concentration, which can result in unpredictable levels of the drug in the body and increase the risk of adverse effects at higher doses [104, 105].

Moreover, first-generation ASMs are characterized by complex drug interactions. For instance, carbamazepine affects the metabolism of other drugs through the cytochrome P450 (CYP) enzyme system, potentially reducing the effectiveness of co-administered drugs and increasing the risk of drug interactions. Similarly, phenobarbital has been shown to induce hepatic CYP enzymes, particularly CYP2C9 and CYP3A4, which can accelerate the metabolism of other antiepileptic drugs such as valproate, carbamazepine, phenytoin, and lamotrigine [106]. This enzymatic induction leads to reduced plasma concentrations of these drugs, potentially diminishing their therapeutic efficacy and increasing the risk of breakthrough seizures or suboptimal seizure control. Phenytoin similarly decreases serum levels of carbamazepine, valproate, and lamotrigine through its induction of CYP3A4 and CYP2C9, which can necessitate dose adjustments and complicated treatment [107]. Despite these limitations, many first-generation ASMs remain in use today due to their proven effectiveness, though their side effects and potential for drug interactions have prompted the development of newer ASMs with improved therapeutic profiles.

#### 3.1.2 Second Generation (1993-2004)

The emergence of second-generation ASMs in the late 20^th^ and early 21^st^ centuries represented a significant advancement in epilepsy treatment, addressing the limitations of first-generation ASMs. Felbamate, the first second-generation ASM, was authorized in 1993. Subsequently, lamotrigine, gabapentin, pregabalin, topiramate, and levetiracetam were developed with distinct improvements over their predecessors. These novel compounds were designed to overcome the shortcomings of earlier ASMs and provide additional options for refractory cases.

The pharmacodynamic profiles of second-generation ASMs significantly expanded the neurochemical landscape of seizure control. Felbamate acts as an NMDA receptor antagonist, while gabapentin and pregabalin modulate voltage-gated calcium channels. Further, tiagabine selectively inhibits GABA reuptake, potentiating GABAergic neurotransmission. The unique mechanism of action of levetiracetam involves modulation of the synaptic vesicle protein 2A, implicated in neurotransmitter release. Topiramate exhibits multiple mechanisms, including sodium channel blockade, GABA potentiation, and glutamate receptor antagonism, allowing for more effective treatment across a broader spectrum of seizure types. This expansion of pharmacological targets and mechanisms of action has facilitated novel approaches for managing drug-resistant epilepsy, offering potential solutions to the challenges posed by pharmacoresistance in patients with refractory seizures.

Second-generation ASMs also exhibit distinct improvements in pharmacokinetics, with optimal half-lives and fewer drug-drug interactions, contributing to improved tolerability and enhanced safety profiles. For instance, pregabalin and gabapentin exhibit minimal interactions with other ASMs, as demonstrated by clinical studies that show their interaction profiles are significantly less complex compared to older drugs [108]. In contrast, first-generation drugs like phenobarbital frequently interact with multiple other ASMs, complicating their use and increasing the likelihood of adverse effects.

Notably, drugs like lamotrigine and levetiracetam have gained widespread clinical adoption due to their broad spectrum of efficacy across various seizure types and improved tolerability profiles compared to some first-generation ASMs. Levetiracetam, in particular, has demonstrated efficacy in patients resistant to sodium channel blockers, and its favorable safety profile has made it valuable for those unresponsive to traditional ASMs [109]. Additionally, beyond epilepsy, these ASMs, like gabapentin and pregabalin, have proven effective in managing neuropathic pain due to their modulation of calcium channels. However, despite these advancements, serious side effects have been reported with second-generation ASMs. For instance, lamotrigine may induce Steven-Johnson syndrome, topiramate is associated with cognitive impairment, and tiagabine may even lead to non-convulsive status epilepticus [110]. Nevertheless, the overall risk-benefit profile of these drugs is generally more favorable than that of first-generation ASMs, particularly in terms of reduced hepatotoxicity and fewer severe idiosyncratic reactions.

#### 3.1.3 Third Generation (2008-2022)

The third generation of ASMs, beginning in the late 20^th^ century, introduced drugs with more targeted mechanisms of action, designed to address specific epilepsy syndromes and improve the treatment of refractory epilepsy. This generation of drugs includes lacosamide, rufinamide, vigabatrin, clobazam, ezogabine/retigabine, perampanel, brivaracetam, everolimus, cannabidiol, cenobamate, fenfluramine, and ganaxolone. These drugs are characterized by novel mechanisms, such as the enhancement of slow inactivation of sodium channels (lacosamide, cenobamate), activation of potassium channels (ezogabine/retigabine), and inhibition of AMPA glutamate receptors (perampanel).

The development of ASMs such as cannabidiol and fenfluramine for conditions like Dravet syndrome underscores a shift toward syndrome-specific treatments. Cannabidiol exerts its effects by modulating the endocannabinoid system, particularly through its influence on type 1 and type 2 cannabinoid receptors, leading to a reduction in excitatory neurotransmission [111]. Fenfluramine, on the other hand, acts as a serotonin and sigma-1 receptor agonist, which not only modulates neurotransmitter release but also influences neuronal excitability through various intracellular signaling pathways [112]. While third-generation ASMs offer promising efficacy and improved tolerability, they are not without side effects. For example, perampanel has been linked to behavioral changes likely due to its modulation of AMPA receptors, which affects mood and cognitive function. However, the selective mechanisms of action of these drugs generally result in fewer systemic interactions compared to earlier-generation ASMs. For example, lacosamide, brivaracetam, and ganaxolone exhibit less antagonistic interactions with other ASMs, reducing the clinical challenges associated with polytherapy that are often required in epilepsy management.

Pharmacokinetic advancements in third-generation ASMs, such as extended half-lives, allow for more convenient dosing schedules and may improve patient adherence. For example, perampanel has a half-life ranging from 50 to 130 hours, which supports once-daily dosing [113]. Similarly, everolimus, with a half-life of 25-35 hours, has shown success in treating seizures associated with tuberous sclerosis complex by inhibiting the mTOR signaling pathway [114]. Overall, third-generation ASMs demonstrate efficacy in treating refractory epilepsy and specific seizure disorders such as Lennox-Gastaut and Dravet syndromes. The precise targeting of the underlying pathophysiological mechanisms in these conditions represents a significant therapeutic advancement.

While each generation introduced new options and mechanisms, many first-generation drugs are still widely used due to their effectiveness. The second and third generations of ASMs introduced more treatment options, particularly for refractory epilepsy and specific syndromes, with newer drugs offering more precise targeting of the underlying pathophysiology. Additionally, these later generations often feature improved pharmacokinetic properties, such as optimal half-life and reduced drug-drug interactions, potentially enhancing patient compliance and treatment outcomes. Nevertheless, the evolution of ASMs across the three generations has not led to a linear improvement in efficacy or adverse effect profiles. Despite some clear advancements, adverse effects remain a persistent challenge. The ongoing development of ASMs aims to further refine these therapies by enhancing their safety profiles and tailoring their mechanisms to address the complex pathophysiology of epilepsy, with the ultimate goal of improving clinical outcomes for patients with diverse and challenging epilepsy syndromes.

### 3.2 Polytherapy in Epilepsy Treatment

The evolution of ASMs from the first to the third generation has significantly expanded the arsenal of therapeutic options available for epilepsy management. Generally, monotherapy is preferred in clinical practice due to regimen simplicity and reduced potential for drug interactions and side effects. However, this approach may not always serve the best interest of the patient, particularly in cases of refractory epilepsy where seizures persist despite the optimal use of a single ASM. This clinical reality has led to the widespread adoption of polytherapy, where two or more ASMs are combined to enhance seizure control.

Monotherapy is the primary treatment for epilepsy, with a second-line ASM being considered if the first one fails [151]. Polytherapy is usually considered after at least two monotherapies have failed, with some guidelines suggesting three trials before switching to polytherapy [152]. Polytherapy combines ASMs with different mechanisms of action to optimize seizure control while minimizing side effects. Key considerations in polytherapy include selecting complementary ASMs, assessing potential pharmacokinetic and pharmacodynamic interactions, and evaluating patient response. Factors such as the type of epilepsy, patient medical history and preferences, current medical conditions, and potential adverse effects must all be carefully weighed when determining the most suitable ASM combination [113].

#### 3.2.1 Mechanisms of Action and Efficacy

Polytherapy in epilepsy management often utilizes the complementary actions of different ASMs to improve treatment effectiveness and seizure control. For absence seizures, combining sodium valproate and ethosuximide has proven particularly effective [153]. These medications work through distinct mechanisms: sodium valproate elevates GABA levels, while ethosuximide targets T-type calcium channels in thalamic neurons, thus offering a more comprehensive absence of seizure management. One study found that 58% of patients under the combination therapy achieved seizure freedom, compared to 53% for ethosuximide alone and 29% for valproate alone [154]. Interestingly, the combination of ethosuximide and valproate has a unique toxicity profile, especially concerning sedation. Despite their additive effects in motor coordination tests, their impact on sedation is distinct. Behavioral studies showed that a higher combined dose is needed to achieve the same level of sedation as the individual drugs, indicating an infra-additive effect [155]. This suggests that patients might experience less sedation with the combination therapy than expected, potentially indicating an increased threshold for this form of toxicity [156]. However, a comprehensive evaluation of the combination's therapeutic effects is still needed to determine if this advantage of reduced sedation leads to a better balance between efficacy and side effects compared to monotherapy.

In managing tonic-clonic seizures, the combination of phenobarbital and phenytoin is an established option. Phenobarbital enhances GABA-mediated inhibition and reduces glutamate release, while phenytoin stabilizes neuronal membranes by inhibiting sodium channels [157]. Notably, before the 1980s, the combined use of phenytoin and phenobarbital was widespread despite limited scientific justification [53, 158]. This empirical approach, although initially lacking robust evidence, laid the groundwork for subsequent studies that confirmed its efficacy. Building on this success, recent research has investigated the potential of three-drug combinations to further improve seizure control in tonic-clonic seizures. A preclinical study found a synergistic effect between phenobarbital, phenytoin, and pregabalin in a mouse model of tonic-clonic seizures. The combination did not cause significant side effects, such as those involving long-term memory, muscular strength, or motor coordination. Further, the combination did not significantly alter the pharmacokinetic profiles of the individual drugs in the brain [159]. This suggests the combination could be potentially applied in clinical practice, offering a new option for epilepsy patients and expanding polytherapy prospects in seizure management.

For patients with refractory epilepsy, the combination of vigabatrin and tiagabine has shown considerable promise. Both drugs work to increase GABA levels but through different mechanisms: vigabatrin inhibits the breakdown of GABA, while tiagabine is a GABA reuptake inhibitor [160]. These drugs affect various targets, including sodium channels and glutamate receptors, providing a wide-ranging impact on neuronal excitability. An isobolographic analysis of the co-administration of vigabatrin and tiagabine in two experimental mice epilepsy models revealed that their combination produced additive effects in the maximal electroshock seizure threshold test and synergistic effects against pentylenetetrazole-induced seizures. Additionally, the combination did not impair motor coordination or neuromuscular tone, suggesting that it could provide effective seizure control with a favorable safety profile [161].

Carbamazepine, which inhibits sodium channels, is often used as a first-line treatment for focal seizures. Its combination with valproate, which increases GABA levels and inhibits sodium and calcium channels [162], addresses multiple aspects of neuronal excitability related to focal seizures for better management of the condition. In one study, 12 out of 17 patients who did not respond to carbamazepine monotherapy showed significant improvement when treated with the combination of valproate and carbamazepine, with six remarkably achieving seizure freedom [163]. In another study, 54 out of 100 patients unresponsive to carbamazepine experienced more than 50% seizure reduction when valproate was added to the treatment regime, with 15 patients becoming seizure-free [164]. Furthermore, it was found that 51.4% of patients experienced more than 50% reduction in seizures with the carbamazepine-valproate combination therapy, compared to 32% with carbamazepine alone [165]. It is important to note, however, that this combination does not necessarily outperform monotherapy for all cases of epilepsy. For instance, a study comparing carbamazepine monotherapy and its combination with valproate found no significant difference in seizure control or toxicity in new-onset epilepsy [166].

The combination of valproate and lamotrigine, which function by inhibiting sodium channels, has also demonstrated promising outcomes in cases of refractory epilepsy [167]. A large European study involving 347 patients found that adding lamotrigine to existing treatments resulted in 47% of patients achieving at least 50% seizure reduction, with the lamotrigine-valproate combination showing the highest response rate at 64% [168]. Another study of 35 patients with refractory epilepsy reported that 51.4% became seizure-free when on lamotrigine-valproate therapy after previous monotherapy attempts had failed [169]. The synergistic effect of lamotrigine and valproate is partly attributed to valproate slowing lamotrigine's clearance, thus increasing its blood concentration [170]. However, this combination requires careful management due to potential adverse effects, including severe tremors and skin rashes [153, 171]. It has been recommended that a slow titration and reduced lamotrigine dosing can help maintain efficacy while minimizing risks [172].

#### 3.2.2 Complexities and Limitations of Polytherapy

The implementation of polytherapy in epilepsy treatment presents numerous challenges for both healthcare providers and patients. One of the primary obstacles in epilepsy treatment is the lack of thorough, evidence-based research on the efficacy of specific ASM combinations. Despite some data supporting various combinations, recent large-scale clinical trials exploring these combinations in detail are scarce. This knowledge gap often forces clinicians to rely on older studies or anecdotal evidence, potentially leading to suboptimal treatment regimens. Many existing studies focus on monotherapy rather than the benefits and risks of combining different drugs. As a result, there is a pressing need for more comprehensive trials to provide clearer guidance on effective and safe ASM combinations. Additionally, the lack of recent data suggests that newer ASMs, which might offer improved safety and efficacy in combination, are not always thoroughly investigated [173]. Thus, addressing this research gap is crucial for developing personalized treatment plans that improve seizure control and patient quality of life.

Another significant challenge is the persistence of older ASMs, such as phenobarbital and phenytoin, in treatment plans. Many patients continue these therapies due to established seizure control, despite the availability of newer options. The reluctance to transition to modern treatments mainly stems from the complexities involved in altering a stable regimen and the potential risks associated with changing medications [174]. However, the long-term use of such ASMs carries additional concerns, including prolonged adverse effects and the need to carefully manage drug interactions [175]. For example, the combination of valproate and lamotrigine requires particularly vigilant monitoring and dosage adjustments. Here, clinical evaluations often prioritize seizure control, sometimes at the expense of thoroughly assessing and managing treatment-related side effects [99].

Once an effective polytherapy regimen is established, reducing the number of medications to mitigate side effects can also be challenging. Patients and healthcare providers may disagree on the necessity or timing of such adjustments, complicating treatment management [176]. Furthermore, although theoretical approaches suggest combining ASMs with different mechanisms of action, the actual effectiveness of these strategies can vary significantly among patients. The lack of experimental data is a significant challenge, leading to potentially beneficial ASM combinations being overlooked in clinical trials due to assumptions about their pharmacological similarities. Moreover, the possibility of additive side effects from various ASM combinations necessitates careful consideration and management in clinical practice [177]. Given these challenges, there is a growing emphasis on developing new ASMs with improved safety profiles that could be effective as monotherapy, aiming to achieve seizure control with a single medication and thus, reducing the complexities associated with polytherapy. Nevertheless, a well-managed polytherapy can be as safe as monotherapy, but clinicians must consider not only the potential synergistic effects of drug combinations but also the possibility of pharmacokinetic and pharmacodynamic interactions that could affect drug levels, efficacy, and toxicity.

### 3.3 Recent Advancements

In this section, we take a closer look at several recently approved ASMs (between 2018 and 2022) whose introductions represent noteworthy advancements in epilepsy therapeutics. The entry of everolimus and cannabidiol to the market in 2018 was followed by cenobamate in 2019 and fenfluramine in 2020. In 2022, the FDA approved for ganaxolone to treat seizures. The recognition of these drugs as ASMs marks a significant development, instilling optimism among patients for effective epilepsy management options offered by these novel drugs.

Everolimus, a rapamycin derivative and selective inhibitor of the mTOR pathway, was approved by the FDA in 2018 for the adjunctive treatment of TSC-induced partial-onset seizures in adults and children. It was originally approved to treat TSC-associated subependymal giant cell astrocytoma and renal angiomyolipoma [114]. The interaction of everolimus with FK506-binding protein 12 (FKBP12) forms a complex that binds to the FKBP12-rapamycin binding domain of mTOR, thus suppressing both mTOR-mediated cell signaling and immune cell proliferation and function [93]. In the context of TSC, the hyperactivation of the mTOR pathway due to mutations in TSC1 or TSC2 genes leads to abnormal cell growth and the formation of brain lesions called tubers which contribute to the development of seizures. Inhibition of mTOR by everolimus reduces the size and number of these tubers, thereby potentially improving seizure control.

Cannabidiol, one of the active cannabinoids derived from the cannabis plant, can be used to treat seizures induced by the Lennox-Gastaut and Dravet syndromes. Furthermore, children and adults with severe refractory epilepsy may also benefit therapeutically from cannabidiol [178, 179]. Additionally, studies have revealed that cannabidiol may be able to reduce the signs and symptoms of anxiety, pain, dystonia, Parkinson's disease, Crohn's disease, and several other conditions [178]. The distinct multimodal analgesic mechanisms of action of cannabinoids allow this class of phytochemicals to act on a variety of pain targets. Nevertheless, a concern regarding cannabidiol administration is its interaction with other drugs, which include opioid analgesics, antidepressants, and other ASMs [180]. For instance, as a result of cannabidiol's inhibition of CYP3A4 and CYP2C19, levels of the active metabolite of clobazam tripled when the two ASMs were co-administered [181].

Cenobamate is one of the most current ASMs developed to treat focal onset seizures in adult patients. Given that it is a carbamate derivative, it has structural features in common with other alkyl-carbamate drugs such as felbamate and retigabine. Hence, these drugs exhibit a similar mechanism of action, acting as positive allosteric modulators of GABA_A_R to enhance inhibitory neurotransmission. Additionally, cenobamate selectively blocks VGSCs in the inactivated state, thus attenuating the persistent, rather than the transient, Na^+^ current [182]. In comparison to other ASMs, adjunctive cenobamate is highly effective in treating refractory focal epilepsy. However, the possibility of developing severe rashes and poor tolerability at higher dosages mitigate its remarkable efficacy [105]. Interestingly, other medications, except for phenytoin, do not affect the bioavailability of cenobamate. However, due to its inhibition of CYP2C19 and conversely, the activation of CYP3A4 and CYP2B6, cenobamate has the potential to interact with and alter the blood concentration of many drugs, including lamotrigine, carbamazepine, and clobazam [183].

Fenfluramine is a phenethylamine with structural similarities to serotonin. It was first employed as an appetite suppressant in the management of obesity until it was removed from the market in 1997 due mainly to reports of valvular heart disease and pulmonary arterial hypertension [147]. It made an unexpected comeback in 2020 for the treatment of individuals two years of age and older suffering from seizures associated with the Lennox-Gastaut and Dravet syndromes. Fenfluramine can increase levels of extracellular serotonin and control neurotransmission by modulating serotonergic and other neurologic receptors [184]. Hence, it is recognized as an effective treatment for pharmacoresistant seizures. Concerning interaction with other ASMs, the combination of stiripentol and clobazam was observed to raise serum fenfluramine levels [185]. Nonetheless, drug interactions between fenfluramine and monoamine oxidase inhibitors may be potentially lethal.

Ganaxolone, known by its brand name Ztalmy, is a 3β-methylated synthetic analog of allopregnanolone that has emerged as a noteworthy neuroactive steroid anticonvulsant [186]. Belonging to the class of neurosteroids, it presents a differentiated pharmacological profile, uniquely modulating both synaptic and extrasynaptic GABA_A_Rs through a specific binding site [187]. Its mechanism of action is focused on normalizing over-excited neurons, thus inhibiting seizure propagation. Its approval by the FDA in 2022, based on results from a multinational phase III trial, underscores its efficacy in reducing seizure frequency, particularly in children and adolescents with cyclin-dependent kinase-like 5 deficiency disorder [150]. Developed by Marinus Pharmaceuticals, the approval of ganaxolone marks a groundbreaking advancement in offering a new therapeutic option in the treatment of this rare neurological disorder.

The recent approval of everolimus, cannabidiol, cenobamate, fenfluramine, and ganaxolone as antiepileptic drugs highlights their unique advantages over earlier generations of ASMs. These newer agents, characterized by their specialized mechanisms of action, have demonstrated enhanced efficacy in managing refractory epilepsy, a domain where traditional ASMs often fall short. Everolimus, a selective inhibitor of the mTOR pathway, directly addresses the pathophysiology of TSC-related seizures by reducing the size and number of tubers, offering a therapeutic benefit not attainable with previous ASMs. Cannabidiol, through its multimodal engagement of various pathways implicated in pain and seizures, provides substantial clinical benefits in treating Lennox-Gastaut and Dravet syndromes. Cenobamate, with its dual mechanism of augmenting GABAergic inhibition and selectively inhibiting inactivated voltage-gated sodium channels, offers superior control of focal onset seizures, outperforming conventional sodium channel blockers. Fenfluramine, reintroduced for its effectiveness in Lennox-Gastaut and Dravet syndromes, modulates serotonergic pathways, delivering potent seizure control in cases where older ASMs have proven inadequate. Ganaxolone, through its modulation of both synaptic and extrasynaptic GABA_A_Rs, represents a novel therapeutic option for cyclin-dependent kinase-like 5 deficiency disorder, a condition with limited treatment options in earlier ASM generations. Together, these drugs signify a considerable advancement in epilepsy therapy, offering more targeted and effective treatments for patients with the most challenging forms of the disorder.

## 4 CHALLENGES IN THE DEVELOPMENT OF ANTISEIZURE MEDICATIONS

Over the years, the search for an effective therapy for epilepsy has resulted in the introduction of several treatment options, comprising medication, surgery, the use of therapeutic devices, and the adoption of a ketogenic diet. Among these, ASMs remain the first treatment of choice for most epileptic patients. In the past three decades, more than a dozen ASMs have been developed with proven effectiveness in controlling seizures of different types. However, challenges remain in developing the next generation of ASMs, primarily, serious adverse reactions to drugs and the existence of seizures that appear to be unresponsive to these drugs. Therefore, it is critical to understand and overcome these obstacles in paving the way forward toward drugs with fewer side effects and a broad antiseizure spectrum.

### 4.1 Adverse Effects of ASMs

Adverse effects can be defined as harmful or unexpected reactions resulting from the administration of a drug [188]. Administration of ASMs has been proven to elicit such effects on multiple systems in the body and thus, is a major issue faced in developing ASMs. In most cases, patients with epilepsy require long-term or even lifelong pharmaceutical treatment, and many have experienced adverse effects in one form or another as a result [189]. Hence, adverse reactions associated with ASMs are considered a key cause of treatment failure [190].

In general, ASMs act by altering neuronal activity in the CNS, and therefore, most of their adverse effects are neurological. Among commonly reported effects include sedation, dizziness, fatigue, headache, ataxia, blurred vision, tremors, cognitive impairment, and behavioral changes [191]. It is discovered that these effects in the CNS are directly linked to drug permeability across the blood-brain barrier (BBB) with several examples of BBB-penetrating ASMs causing the aforementioned undesired [192, 193]. Nonetheless, it is important to note that BBB permeability is crucial for the therapeutic efficacy of many ASMs as their targets are in the brain. The challenge lies in achieving a balance between sufficient CNS penetration for therapeutic effect and minimizing adverse effects. Factors such as drug selectivity for specific targets, regional brain distribution, and dose-dependent effects all contribute to the complex relationship between BBB permeability and both therapeutic and adverse effects of ASMs [194]. Ongoing research aims to optimize this balance through various strategies, including targeted drug delivery and the development of more selective compounds [195].

While the new generation of ASMs had similar efficacy to older ASMs, they exhibit fewer drug-drug interactions and have much-improved side effect profiles as summarized in Table (**1**) [129]. Although most of these adverse reactions are tolerable, there are, however, some that are life-threatening. For example, felbamate has been linked to the development of aplastic anemia and hepatic failure [196], while carbamazepine may induce Stevens-Johnson syndrome and toxic epidermal necrolysis, which are rare but severe cutaneous adverse reactions [197]. In short, the adverse effects of ASMs cannot be overlooked and should be considered as a primary concern in the development of novel ASMs.

### 4.2 Drug-resistant Epilepsy

The enigma of drug-resistant epilepsy (DRE), commonly known as refractory epilepsy, has spurred the quest for innovative medications that address this persistent challenge. Despite concerted efforts by researchers worldwide, a definitive solution to this complex puzzle remains elusive. According to the International League Against Epilepsy (ILAE), drug-resistant epilepsy is defined as “failure of adequate trials of two tolerated and appropriately chosen and used ASM schedules (whether as monotherapies or in combination) to achieve sustained seizure freedom” [198]. Simply put, individuals with epilepsy who continue to experience seizures unresponsive to ASMs are diagnosed with DRE. Remarkably, these individuals also face elevated risks of mortality and mental health disorders compared to other epilepsy patients [199]. Given the gravity of this situation, there is an urgent need for the development of more efficacious treatments to address the challenges of DRE.

The emergence of refractory epilepsy has perplexed researchers for years and is now recognized as a complex and multifaceted problem. In recent times, several hypotheses have been presented to explain the underlying mechanism of pharmacoresistance. According to the pharmacokinetic hypothesis, overexpression of efflux transporters in peripheral organs lowers ASM plasma levels in DRE patients, thereby limiting the amount of ASM that may cross the BBB [200]. Closely related to this, the drug transporter hypothesis proposes that DRE is caused by increased expression of multidrug efflux transporters in the BBB, which limits ASM uptake in the brain [201]. The neural network hypothesis proposes that seizure-induced degeneration and reconfiguration of the neural network suppresses the endogenous antiseizure system, reducing the efficacy of ASMs [200, 202]. The intrinsic severity hypothesis meanwhile, considers pharmacoresistance as a characteristic of epilepsy that is associated with the severity of the disease [203]. On the other hand, the gene variant hypothesis postulates that pharmacokinetic and pharmacodynamic profiles of ASMs are affected by polymorphisms or gene variations, resulting in inherent pharmacoresistance [200, 204]. Lastly, the target hypothesis suggests that DRE is caused by molecular alterations in therapeutic targets, rendering ASMs ineffective [205, 206]. Nevertheless, none of these hypotheses can alone adequately explain the neural underpinnings of DRE [207].

The BBB serves to maintain and protect the brain's microenvironment by acting as a physical and metabolic barrier between the CNS and the peripheral circulation. This barrier is critical for maintaining brain homeostasis [208, 209]; therefore, dysfunction of the BBB has been linked to several neurological disorders, including epilepsy, multiple sclerosis, stroke, brain tumors, and Alzheimer's disease [210-212]. Hence, pharmacoresistance in epilepsy is believed to be partly caused by BBB dysfunction and the extravasation of serum proteins into the brain. The loss of BBB integrity can lead to an influx of serum albumin into the brain, which subsequently binds to ASMs. As a result, the concentration of free, non-protein-bound ASMs in the brain decreases, affecting their pharmacological activity [213-215].

Insights from past studies have deepened our understanding of the intricate mechanisms underlying DRE. Crucially, they provide a solid foundation for the development of ASMs in effectively addressing this increasingly prevalent and complex neurological disorder. With DRE posing a growing challenge, these findings have the potential to reshape the landscape of epilepsy treatment, offering renewed hope for individuals affected by this condition.

## 5 ADVANCING ANTISEIZURE MEDICATIONS: IDEAL CHARACTERISTICS AND POTENTIAL CANDIDATES

Considering that the majority of existing ASMs do not satisfy contemporary medical needs, there is a pressing need to identify feasible therapeutic drugs with favorable pharmacokinetic properties. Here we discuss the ideal characteristics that a drug should possess to function effectively as an ASM, taking into account the challenges mentioned above.

### 5.1 Ideal Characteristics of Antiseizure Medications

An ideal ASM must, first and foremost, be more efficacious against different types of seizures, particularly refractory epilepsy. According to current estimates, only about two-thirds of individuals who are on ASM therapy manage to successfully control their seizures, with the other third showing treatment resistance [216]. Therefore, the main goal of newly introduced ASMs should be to completely stop seizures and enable patients to achieve continued seizure freedom.

When an epileptic condition that results in chronic hyperexcitability first appears, it is referred to as epileptogenesis [217]. Both spontaneous and injury-related occurrences are possible. The term also refers to the process through which seizures in patients with recurrent epilepsy worsen and occur more often. The two most prominent cases of adult epileptogenesis are those that occur after a stroke or a traumatic brain injury [218, 219]. The majority of currently available ASMs target the GABAergic system or voltage-gated ion channels to lower excessive neuronal activity in the brain during seizures. However, they do not tackle the underlying brain disorder and thus, are not regarded as antiepileptogenic or disease-modifying [220]. Therefore, drugs with the ability to modify the disease or prevent epileptogenesis are considered attractive ASM candidates.

In addition to treating epilepsy, an ASM should ideally be effective in treating non-epileptic CNS conditions, including bipolar disorder, TSC, and autism spectrum disorder (ASD). The frequent co-occurrence of mood disorders with epilepsy is a widely observed phenomenon. For instance, it is reported that those with epilepsy are more prone to having bipolar symptoms [221]. Furthermore, there exists a complicated relationship between epilepsy, ASD, and TSC. Epilepsy is estimated to impact 80% of TSC patients and is also regarded as a potential risk factor for ASD in TSC patients, particularly when epilepsy manifests in infancy and is accompanied by infantile spasms [222, 223]. Moreover, seizures are the most common neurologic complication in ASD, closely interlinking epilepsy and autism. Additionally, dysregulation of GABA neurotransmission is regarded as the physiological link between epilepsy and ASD in TSC patients [224]. Consequently, ASMs that can be administered to treat non-epileptic CNS illnesses are highly significant in the landscape of epilepsy management due to their therapeutic value.

Finally, greater tolerability with reduced adverse effects must be made an important criterion for any potential drug for further development into an ASM. The tolerability profile of a drug is often more crucial than its therapeutic efficacy since it has a significant impact on the adherence of patients to their treatment, which ultimately determines the success of the prescribed therapy. Unsurprisingly, poor tolerability is recognized as one of the most common reasons for changing prescriptions among epileptic patients [225, 226]. Generally, newer ASMs are more effective due to their ease of use. Rapid titration, linear pharmacokinetics, little or no drug interactions, and once-daily dosing are all favorable characteristics of medications with high acceptance by patients [227].

### 5.2 Potential ASMs Under Clinical Trial

The EILAT Conference on New Antiepileptic Drugs and Devices, held biennially, is a leading platform in epilepsy research, facilitating collaboration among scientists, clinicians, and experts on novel antiseizure treatments. The conference highlights promising candidates with unique mechanisms of action for treating neurological disorders and serves to accelerate the development of ASMs based on multidisciplinary insights. Table **2** lists examples of potential compounds currently under various stages of clinical trials as ASMs, as described in the preceding progress reports of the Eilat Conferences [186, 228-230].

Lorcaserin, originally an appetite suppressant, was withdrawn from the market eight years post-FDA approval due to potential cancer risks. An agonist of the 5-HT2C receptor, it was then repurposed for epilepsy treatment. Remarkably, lorcaserin was effective in Dravet syndrome models and progressed to a phase 3 trial for efficacy and safety assessments in individuals with Dravet syndrome [231]. Another compound currently undergoing phase 3 trial for Dravet and Lennox-Gastaut syndromes is soticlestat (TAK-935), a first-in-class experimental ASM that is a potent and highly selective inhibitor of cholesterol 24-hydroxylase. It presents a promising strategy for addressing these forms of epilepsy by targeting the abnormal cholesterol metabolism implicated in developmental and epileptic encephalopathies associated with seizures [186, 232].

The emphasis on precision medicine and the exploration of non-traditional targets highlights the evolving strategies in epilepsy management. This is demonstrated by the development of compounds such as NBI-921352 (formerly XEN901), PRAX-562, and XEN496, each designed to target distinct genetic or molecular factors associated with epilepsy. For instance, XEN496 which is currently in a phase III trial, targets the potassium voltage-gated channel subfamily Q member 2 developmental and epileptic encephalopathy (KCNQ2-DEE), a specific epilepsy syndrome [233].

ETX-020155, ENX-101, and darigabat form a notable cluster among the listed compounds, all acting as positive allosteric modulators of GABA_A_R. ETX-020155, currently in phase 1, exhibits potential as an antiepileptic, antidepressant, and antianxiety agent. ENX-101, in phase 1b/2, represents a precision-targeted GABA_A_R modulator, targeting the α2, α3, and α5 subunits to elicit therapeutic effects in patients with focal epilepsy while minimizing undesired side effects [234]. Similarly, darigabat (CVL-865) selectively targets α2, α3, and α5 subunits. Undergoing phase 2 trials, it shows promise as a potential treatment for anxiety and epilepsy, with structural variations from classical benzodiazepines to minimize adverse events associated with α1-containing receptors [235]. As such, this class of drugs signifies a trend toward nuanced modulation of neurotransmission for enhanced efficacy with minimized side effects in treating neurological disorders.

The compounds mentioned above exhibit promising characteristics that align with the criteria of an ideal ASM. Their demonstrated efficacy in treating various forms of refractory epilepsy, including focal seizures associated with developmental and epileptic encephalopathies, Lennox-Gastaut syndrome, and Dravet syndrome, positions them as valuable additions to the epilepsy treatment options. Their success in addressing uncommon epilepsies contributes to filling a critical gap in the market for drugs that can effectively manage diverse types of epilepsy, especially in pediatric patients with limited treatment choices. Moreover, their potential applications extend beyond epilepsy, showcasing therapeutic versatility that aligns with the evolving trend of developing medications with multiple functions. Furthermore, these compounds possess favorable tolerability profiles with reduced drug interactions and have a less negative impact on cognitive function compared to previous ASMs. The emphasis on tolerability and cognitive impact reflects a patient-centric approach, making these compounds not only effective in addressing seizures but also considerate of the overall well-being of patients in terms of reduced adverse effects. In summary, these compounds, with their efficacy, versatility, and improved tolerability, hold promise as potential ASMs in the future, offering a comprehensive solution to the complex challenges associated with epilepsy treatment.

## 6 FUTURE PROSPECTS

The majority of ASMs, as shown in Table (**1**), are typically associated with adverse effects such as somnolence, fatigue, and dizziness. Hence, much effort has been put into exploring potential drugs possessing anticonvulsant properties with minimal side effects. These newly developed drugs can be classified into two groups: those with completely new chemical structures and those with structural modifications to current ASMs [236]. In general, the discovery of a lead compound with the desired biological activity is the first step in the development of a new drug. Following that, derivatives of the lead compound with enhanced pharmacokinetic or pharmacodynamic properties can be generated.

As the discovery of new drugs is time-intensive, most drugs are created by modifying existing ASMs to take advantage of the desirable attributes of the parent compound such as proven efficacy. For instance, valproic acid is an ASM with a simple molecular structure that is widely used to treat various types of epilepsies. However, its use in pregnant women and children has been restricted primarily due to its severe side effects, hepatotoxicity, and teratogenicity [237, 238]. Thus, valproic acid has been chemically modified to produce compounds with improved anticonvulsant action and reduced teratogenicity [239]. Valproic acid derivatives such as valpromide and sodium divalproate offer distinct advantages over valproic acid, with valpromide exhibiting mood-stabilizing effects in bipolar disorder patients alongside its antiepileptic properties, while sodium divalproate was specifically designed to reduce gastrointestinal side effects [240]. These modifications not only enhance therapeutic benefits but also minimize adverse effects, making these derivatives potentially safer and more effective options for clinical use compared to valproic acid. In addition, everolimus, which is derived from rapamycin, has superior pharmacokinetic properties compared to rapamycin. This includes improved absorption, increased oral bioavailability, faster attainment of steady-state levels after administration, and quicker elimination after withdrawal [241]. Both rapamycin and everolimus have been shown to reduce the activation of microglia while inhibiting the activation of markers associated with inflammation. However, in seizure and neuroinflammation models, everolimus demonstrated better efficacy at inhibiting the iNOS and mTOR signaling pathways [242].

Among the 28 ASMs that have been approved (Table **1**), about a third increase the inhibitory effects of GABA by either modulating GABA_A_R activity or by affecting GABA availability. Possessing at least seven allosteric and three orthosteric binding sites, GABA_A_R continues to be an attractive target of new ASMs. Numerous studies have demonstrated that a decrease in GABA_A_R expression in the brain contributes to the development of focal epilepsy with or without secondary generalization. Further, a marked reduction in benzodiazepine binding sites has been attributed to mesial temporal lobe epilepsy, the most common type of refractory focal epilepsy [243].

As mentioned earlier, several prospective therapeutic candidates that target GABA_A_R are now undergoing clinical trials, including ETX-020155, ENX-101, and darigabat [244, 245]. It is interesting to note here that isoguvacine, an agonist of GABA_A_R and an inhibitor of acetylcholinesterase [246], has also emerged as a prospective ASM. Some studies on its anticonvulsant profile have demonstrated its inhibitory effects on generated seizures [247-249]. Moreover, it may also be effective in treating other neurological disorders such as autism, as it has been proven to alleviate tactile hypersensitivity and improve brain microcircuit function while avoiding the risks of direct brain stimulation [250]. The zwitterionic nature of isoguvacine provides stability and reduces systemic side effects, though it also limits BBB permeability, thus restricting its direct effects on the CNS. However, by targeting GABA_A_Rs located in peripheral neurons or in specific brain regions where the BBB is less restrictive, such as the circumventricular organs, isoguvacine can still modulate seizure activity. Additionally, recognizing its pharmacokinetic limitation for efficient seizure control, we have designed ester prodrug derivatives of isoguvacine capable of crossing the BBB. Once inside the brain, the prodrug is expected to convert into the active isoguvacine that will exert its antiseizure effects locally without causing side effects outside the CNS.

Although the pharmacological effects of isoguvacine have been studied in animals, the administration of isoguvacine in humans has not been documented. Due to the lack of research on its interaction with plasma proteins, its biological efficacy is unknown. Such studies are important since the bioavailability, distribution, metabolism, and elimination of a drug are all influenced by its interaction with plasma transport proteins. Also, understanding and predicting pharmacological features such as *in vivo* efficacy and potential adverse effects requires the assessment of such interactions [251]. Given the significance of isoguvacine in the treatment of epilepsy and other CNS diseases, the authors are actively engaged in exploring intermolecular interactions involving isoguvacine, human transport proteins, and GABA_A_Rs. This also includes the synthesis of novel derivatives of isoguvacine with improved pharmacokinetics to establishing isoguvacine as a potential ASM.

## CONCLUSION

Epilepsy is a common neurological condition that is expected to remain a public health concern for years to come. Clinically, ASMs continue to be the main therapeutic intervention for epileptic patients. Elucidation of the conventional and unique mechanisms of action of ASMs as well as characterization of their interactions with receptors have helped us to better understand the therapeutic impact of these drugs. This understanding is pivotal in improving epilepsy management and patient outcomes, particularly when assessing the options of monotherapy and polytherapy, together with their associated complexities and limitations. While there is no denying that recently developed ASMs are more successful and tolerable than previous ASMs, they do still have significant drawbacks. Therefore, research is focused on discovering and developing novel ASMs that are efficient, safe, and elicit fewer adverse effects. Nevertheless, the challenges faced in developing novel drugs must also be addressed. Within this context, the discovery of new ASMs through chemical modifications of existing drugs is an attractive strategy due to its obvious benefits. Finally, the focus on GABA_A_R modulation in developing new ASMs highlights the continuing importance of this mechanism in terms of its therapeutic application.

## Figures and Tables

**Fig. (1):**
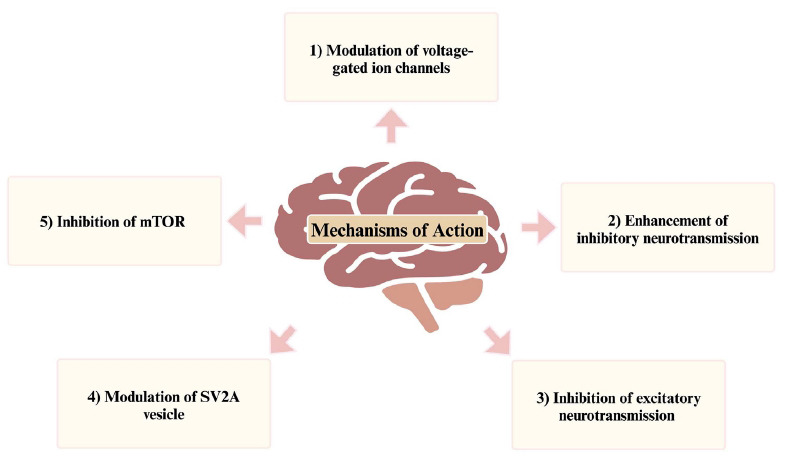
An overview of the mechanisms of action employed by conventional and newer ASMs.

**Fig. (2):**
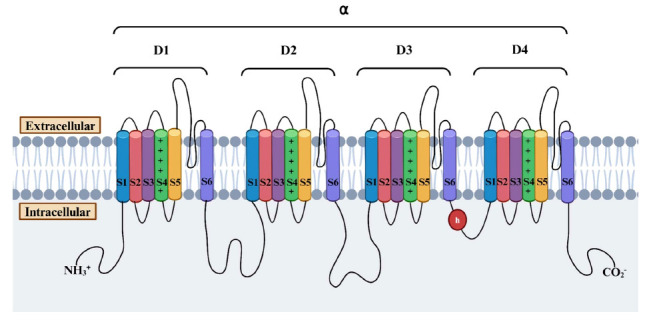
Schematic representation of voltage-gated ion channels showing their intricate architecture and key features. The α-subunit serves as the core of the channel and is composed of four homologous domains (D1-D4), each housing six transmembrane segments (S1-S6). The S1-S4 segments form the voltage-sensing module that detects changes in membrane potential, while the selectivity filter consists of the pore loops between the S5 and S6 segments.

**Table 1:** Overview of FDA-approved ASMs for epilepsy management.

**Classifi-cation**	**ASM**	**Mechanism of Action**	**Indication**	**Pharmacokinetic Properties**		**Common Adverse Effects**	**References**
				**Half-life (hours)**	**Drug-Drug Interactions**		
First Generation (1912-1981)	Phenobarbital	Enhances GABAergic inhibition	Generalized tonic-clonic seizures, focal seizures	70-140	● Decreases serum concentrations of valproate, carbamazepine, phenytoin, and lamotrigine	Sedation, somnolence, dizziness	[115, 116]
	Phenytoin	Blocks voltage-gated sodium channels	Generalized tonic-clonic seizures, focal seizures	7-42	● Decreases serum concentrations of carbamazepine, valproate, and lamotrigine	Sedation, dizziness, ataxia, gingival hyperplasia	[110, 117, 118]
	Ethosuximide	Blocks T-type calcium channels	Absence seizures	30-60	● Serum concentration increased by valproate ● Serum concentration decreased by phenytoin	Sedation, nausea	[54, 108, 119]
	Diazepam	Enhances GABAergic inhibition	Acute seizures, status epilepticus	20-70	● Serum concentration decreased by CYP3A4 inducers (*e.g*., rifampin)	Sedation, somnolence, fatigue, ataxia, muscle weakness	[120, 121]
	Carbamazepine	Blocks voltage-gated sodium channels	Partial seizures, trigeminal neuralgia	18-65	● Decreases serum concentrations of valproate, lamotrigine, topiramate, and oxcarbazepine ● Serum concentration increased by CYP3A4 inhibitors (*e.g*., erythromycin, ketoconazole)	Dizziness, nausea, ataxia, blurred vision	[108, 122, 123]
	Clonazepam	Enhances GABAergic inhibition	Absence seizures, myoclonic seizures	30-40	● Serum concentration decreased by CYP3A4 inducers (*e.g*., rifampin)	Sedation, somnolence, dizziness	[124, 125]
	Valproate	Enhances GABAergic inhibition, sodium channel blockade	Generalized tonic-clonic seizures, absence seizures	9-16	● Increases serum concentrations of phenobarbital, carbamazepine-10,11-epoxide, and lamotrigine ● Serum concentration decreased by carbamazepine and phenytoin	Somnolence, dizziness, tremor, hair loss, weight gain	[110, 126]
Second Generation (1993-2004)	Felbamate	NMDA receptor antagonist enhances GABAergic inhibition	Lennox-Gastaut syndrome, focal seizures	11-23	● Increases serum concentrations of phenytoin, valproate, and carbamazepine-epoxide ● Serum concentration decreased by carbamazepine and phenytoin	Somnolence, dizziness, nausea, insomnia, weight loss, blurred vision, hepatic failure	[119, 127, 128]
	Gabapentin	Modulates α2δ-1 subunit of Ca^2+^ channel	Partial seizures, neuropathic pain	5-9	● Minimal interactions with other ASMs	Sedation, dizziness, weight gain	[127-129]
	Lamotrigine	Blocks voltage-gated sodium channels	Generalized tonic-clonic seizures, focal seizures	20-40	● Decreases the serum concentration of levonorgestrel ● Serum concentration increased by valproate ● Serum concentration decreased by carbamazepine and phenytoin	Somnolence, dizziness, insomnia, rash	[127-129]
Second Generation (1993-2004)	Topiramate	Blocks voltage-gated sodium channels, enhances GABAergic inhibition	Generalized tonic-clonic seizures, focal seizures	20-30	● Increases serum concentration of phenytoin	Somnolence, dizziness, paresthesia, anorexia, weight loss, fatigue, nervousness, psychomotor disturbance, memory and cognitive impairment, mood changes	[127-129]
	Tiagabine	Inhibits GABA reuptake	Partial seizures	5-9	● Serum concentration decreased by enzyme-inducing ASMs	Sedation, dizziness, fatigue, weight loss, tremor	[119, 127, 128]
	Levetiracetam	Modulates synaptic vesicle protein SV2A	Generalized tonic-clonic seizures, focal seizures	6-8	● Serum concentration decreased by enzyme-inducing ASMs	Sedation, dizziness, somnolence, behavioral and psychiatric problems	[127-129]
	Oxcarbazepine	Blocks voltage-gated sodium channels	Focal seizures	7-12	● Decrease serum concentrations of phenytoin and carbamazepine ● Serum concentration increased by valproate	Sedation, dizziness, hyponatremia, weight gain, rash	[127-129]
	Zonisamide	Blocks voltage-gated sodium channels, T-type calcium channel blockade	Focal seizures	50-70	● Serum concentration decreased by enzyme-inducing ASMs	Somnolence, dizziness, weight loss, rare nephrolithiasis	[119, 127, 128]
	Pregabalin	Modulates α2δ-1 subunit of Ca^2+^ channel	Focal seizures, neuropathic pain	5-7	● Minimal interactions with other ASMs	Somnolence, dizziness, ataxia, blurred vision, weight gain, asthenia	[127, 128, 130]
Third Generation (2008-2022)	Lacosamide	Enhances slow inactivation of sodium channels	Focal seizures	12-16	● Serum concentration decreased by enzyme-inducing ASMs	Somnolence, dizziness, headache, nausea, diplopia	[128, 131, 132]
	Rufinamide	Prolongs sodium channel inactivation	Lennox-Gastaut syndrome, focal seizures	8-12	● Serum concentration increased by valproate	Somnolence, dizziness, headache, ataxia, blurred vision, fatigue, nausea, rash	[127, 128, 133]
	Vigabatrin	Irreversibly inhibits GABA transaminase	Refractory complex partial seizures, infantile spasms	4-7	● Decreases serum concentrations of phenytoin	Somnolence, dizziness, headache, fatigue, blurred vision, memory impairment, weight gain, abnormal coordination	[128, 129, 134]
	Clobazam	Enhances GABAergic inhibition	Lennox-Gastaut syndrome, seizures associated with Dravet syndrome	36-42	● Serum concentration increased by valproate and cannabidiol	Somnolence, ataxia, aggression, fatigue, and insomnia	[127, 135, 136]
	Ezogabine/ Retigabine	Activates KCNQ2/KCNQ3 potassium channels	Focal seizures	8-10	● Minimal interactions with other ASMs	Somnolence, dizziness, confusional states, fatigue, vertigo, tremor, ataxia, diplopia	[127, 137, 138]
	Perampanel	AMPA glutamate receptor antagonist	Focal seizures	50-130	● Serum concentration decreased by enzyme-inducing ASMs ● Decreases the effectiveness of levonorgestrel-containing oral contraceptives	Somnolence, dizziness, psychomotor impairment, aggression, ataxia, blurred vision, irritability, dysarthria	[127-129]
Third Generation (2008-2022)	Brivaracetam	Modulates synaptic vesicle protein SV2A	Focal seizures	6-11	● Increases serum concentration of carbamazepine-10,11-epoxide ● Serum concentration increased by cannabidiol ● Serum concentration decreased by enzyme-inducing ASMs (moderate reduction)	Sedation, somnolence, dizziness, fatigue, nausea, nasopharyngitis	[128, 139, 140]
	Everolimus	Inhibits mTOR signaling pathway	Refractory focal-onset seizures associated with tuberous sclerosis complex	25-35	● Serum concentration increased by P-gp and CYP3A4 inhibitors ● Serum concentration decreased by enzyme-inducing ASMs	Dizziness, headache, weight loss, fever, diarrhea, edema, peripheral edema, fatigue, nausea, rash, stomatitis	[128, 139, 141-143]
	Cannabidiol	Modulates endocannabinoid system	Lennox-Gastaut syndrome, Dravet syndrome	10-17	● Increases serum concentrations of clobazam and its active metabolites	Somnolence, nausea, diarrhea, fatigue, hepatic abnormalities	[128, 139, 144]
	Cenobamate	Enhances voltage-gated sodium channel inactivation	Focal seizures	50-60	● Decreases serum concentrations of carbamazepine, phenytoin, and phenobarbital ● Serum concentration decreased by phenytoin	Somnolence, dizziness, headache, fatigue, nausea	[139, 145, 146]
	Fenfluramine	Serotonin and sigma-1 receptor agonist	Dravet syndrome	20	● Serum concentration increased by the combination of stiripentol and clobazam ● Serum concentration decreased by CYP3A4 inducers ● Risk of serotonin syndrome when used with other serotonergic drugs	Nasopharyngitis, diarrhea, pyrexia, reduced appetite, fatigue	[139, 147, 148]
	Ganaxolone	Positive allosteric modulator of GABA receptors	Refractory status epilepticus, seizures associated with cyclin-dependent kinase-like 5 deficiency disorder	34	● Minimal interactions with other ASMs	Somnolence, dizziness, headache, fatigue, nausea, pyrexia, hypersecretion of saliva, seasonal allergies	[149, 150]

**Table 2:** Drug candidates under clinical trial for the treatment of seizures.

**Drug Candidate**	**Phase**	**Mechanism of Action**	**Uses**
ETX-020155	1	Positive allosteric modulator of GABA_A_R	Antiepileptic, antidepressant, antianxiety
Nab-sirolimus (ABI-009)	1	mTOR inhibitor	Evaluation in pediatric patients with surgically refractory epilepsy
ENX-101	1b/2	Positive allosteric modulator of GABA_A_R (selective for α2, α3, α5 subunits)	Adjunctive therapy in patients with focal epilepsy
Darigabat (CVL-865)	2	Positive allosteric modulator of GABA_A_R (selective for α2, α3, α5 subunits)	Potential treatment for anxiety and epilepsy
LP352 (Bexicaserin)	2a	5-HT2C receptor superagonist	Treatment for developmental and epileptic encephalopathies
NBI-921352 (XEN901)	2	Sodium channel inhibitor (NaV1.6 specific)	Treatment for SCN8A developmental and epileptic encephalopathy (phase 2 failed for adult focal onset seizures)
PRAX-562	2	Preferential inhibitor of persistent sodium current	Treatment for developmental and epileptic encephalopathies
Rozanolixizumab	2	FcRn inhibitor (monoclonal antibody)	Treatment for leucine-rich glioma inactivated 1 autoimmune encephalitis
Lorcaserin	3	5-HT2c receptor agonist	Potential treatment for Dravet syndrome
Soticlestat (TAK-935)	3	Cholesterol 24-hydroxylase blocker	Treatment for seizures in Dravet syndrome
STK-001	3	Antisense oligonucleotide (ASO) targeting SCN1A gene	Potential disease-modifying therapy for Dravet syndrome
XEN496	3	KCNQ2 channel opener	Precision medicine for KCNQ2-DEE pediatric epilepsy
XEN1101	3	K_v_7.2/K_v_7.3 potassium channel opener	Adjunctive treatment for focal onset seizures

## LIST OF ABBREVIATIONS

AMPA: α-Amino-3-hydroxy-5-methyl-4-isoxazole Propionic Acid
ASD: Autism Spectrum Disorder
ASMs: Antiseizure Medications
BBB: Blood-brain Barrier
CNS: Central Nervous System
CYP: Cytochrome P450
DRE: Drug-resistant Epilepsy
EMA: European Medicines Agency
FDA: US Food & Drug Administration
FKBP12: FK506-binding Protein 12
GABA: γ-Aminobutyric Acid
GABA_A_R: γ-Aminobutyric Acid Type A Receptor
iGluRs: Ionotropic Glutamate Receptors
ILAE: International League Against Epilepsy
INaP: Persistent Sodium Current
KCNQ: K_v_7 Potassium Channel Family
K_v_: Voltage-gated Potassium Channels
LVA: Low Voltage-activated
mTOR: Mechanistic Target of Rapamycin
mTORC1: mTOR Complex 1
mTORC2: mTOR Complex 2
NMDA: N-methyl-D-aspartate
SV2A: Synaptic Vesicle Glycoprotein 2A
TCA: Tricarboxylic Acid
TSC: Tuberous sclerosis complex
VGCCs: Voltage-gated Calcium Channels
VGSCs: Voltage-gated Sodium Channels
